# Disrupted brain topological network and its association with clinical features in posterior cortical atrophy

**DOI:** 10.1093/braincomms/fcag014

**Published:** 2026-01-20

**Authors:** Min Chu, Qianqian He, Min Cui, Hong Ye, Caishui Yang, Liyong Wu

**Affiliations:** Department of Neurology, Xuanwu Hospital, Capital Medical University, Beijing 100053, China; Department of Neurology, Xuanwu Hospital, Capital Medical University, Beijing 100053, China; Department of Neurology, Xuanwu Hospital, Capital Medical University, Beijing 100053, China; Department of Neurology, Xuanwu Hospital, Capital Medical University, Beijing 100053, China; Faculty of Psychology, Beijing Normal University, Beijing 100875, China; Beijing Key Laboratory of Cognitive Intelligence for Elderly Brain Health, Beijing Normal University, Beijing 100875, China; Department of Neurology, Xuanwu Hospital, Capital Medical University, Beijing 100053, China; Department of Geriatric, National Clinical Research Center for Geriatric Diseases, Beijing 100053, China

**Keywords:** posterior cortical atrophy, topological network, graph theory, diffusion tensor imaging

## Abstract

The characteristics of structural brain topological network alterations and their correlation with clinical features in posterior cortical atrophy (PCA) remain elusive. This study aims to explore the structural topological network alterations and their correlation with clinical features in PCA. Thirty-four patients with PCA and 34 healthy controls were enrolled in this cross-sectional study and underwent diffusion tensor imaging, structural MRI and neuropsychological assessment. The graph theory method was applied to capture the individual structural properties of the network. Partial correlation analysis was conducted to investigate the clinical relevance of the network properties. The global metrics of the structural and topological network are altered in the PCA group. The nodal metrics were changed in the occipital, parietal, temporal and frontal lobes. Global network metrics are associated with cognition and disease severity. For the node associated with apraxia, finger agnosia, left-right disorientation and agnosia for colour, face and object, we observed a similar but not identical nodal distribution, which was mainly distributed in the parietal cortex. Notably, we observed frontal nodes, including the orbital gyrus, contribute to the left-right disorientation and colour agnosia, and the rectus contributes to the object agnosia. This study helps us understand the underlying mechanism of the symptom network in PCA and provides a promising biomarker for PCA.

## Teaser

Chu *et al*. demonstrated altered global and nodal structural network metrics in patients with posterior cortical atrophy. These network changes, distributed across parietal, occipital, temporal, and frontal lobes, were associated with cognition, disease severity, and specific visuospatial and perceptual symptoms.

## Graphical Abstract

**Graphical Abstract:**
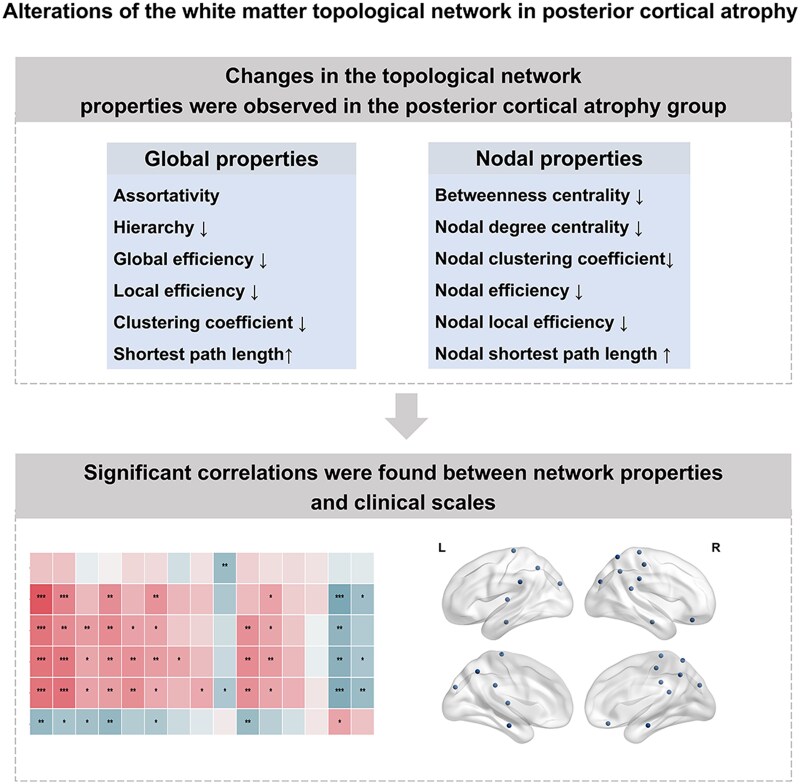

## Introduction

Posterior cortical atrophy (PCA), a rare clinical syndrome initially characterized in 1988, typically manifests with degeneration of posterior brain regions, including the occipital, parietal, and temporal cortices.^1^ The majority of the PCA cases are caused by Alzheimer’s disease (AD) pathology, and PCA is recognized as an atypical form of AD.^2,3^ The main symptoms of PCA include dysfunction and progressive decline in higher-visual object and space processing, manifested as visuospatial deficits, alexia, agnosia, and apraxia.^4,5^ Previous studies have confirmed that patients with PCA exhibit atrophy, hypometabolism, or hypoperfusion in the occipitoparietal and temporal areas,^6-10^ as well as damage to the integrity of posterior white matter tracts.^8,11-14^ In addition, small-sample studies in PCA have reported reduced connectivity in the visual and dorsal attention networks and tau protein deposition.^8,14-17^

Contemporary neuroimaging research increasingly utilizes graph-theoretical approaches, where brain networks are represented as node-edge graphs (nodes denoting neural regions and edges indicating interregional connectivity). This framework enables quantitative characterization of complex brain network topologies.^18-20^ Graph theory metrics such as nodal clustering coefficient, nodal shortest path length, nodal efficiency, and nodal degree can reflect different aspects of network features, which are significant not only for understanding the whole-brain network imbalance in PCA but also for exploring the potential mechanisms of the disease.^19,21^ While prior fMRI studies have demonstrated widespread functional network disruption in PCA,^17^ structural network topology may offer some unique insights. Diffusion tensor imaging (DTI) enables *in vivo* reconstruction of white matter fibre tracts, providing a direct measure of anatomical connectivity that reflects information flow between brain regions more accurately than other neuroimaging modalities.^19,20,22^ However, alterations and clinical significance in the structural topological network of PCA patients remain unclear; whether the nodes outside the occipital–parietal–temporal cortex contribute to the visual symptoms needs to be elucidated.

This study included a group of patients with PCA and healthy controls, and both groups underwent DTI and T1-weighted imaging. First, we calculated the structural and metabolic pattern of the PCA group, then we constructed brain topological networks to identify the characteristics of structural network alterations in PCA patients and evaluate the correlation with clinical features. We hypothesize that the brain structural topological network was disrupted in the PCA group and related to the clinical features; brain regions outside the occipital–parietal–temporal regions might also contribute to the clinical outcome.

## Materials and methods

### Participants

This study was conducted in accordance with the Declaration of Helsinki, and written informed consent was obtained from all participants or their legal guardians. The Ethics Committee on Human Clinical Research at Xuanwu Hospital, Capital Medical University, approved the study protocol. From 2017 to 2024, 34 patients with PCA and 34 healthy controls (HC) were recruited from the Neurology Department. PCA diagnosis followed the established 2017 criteria,^4^ with vision-related cognitive deficits as the primary symptom, rather than executive dysfunction, memory, or language. All PCA cases were amyloid-positive (^18^F-AV45 PET positive).

Cognitively normal control (NC) participants met education-adjusted cut-off scores on cognitive tests: Mini-Mental State Examination (MMSE >19, >22 and >24 for no formal education, primary school and secondary school or higher, respectively) and Montreal Cognitive Assessment (MoCA >13, >19 and >24 for the same education levels). Additionally, NC subjects had a Clinical Dementia Rating (CDR) sum of boxes score of 0.^23-25^

All participants underwent thorough ophthalmic evaluations to confirm that corrected visual acuity did not affect reading ability. Exclusion criteria included visual impairments, depression, severe psychiatric disorders, epilepsy, cerebrovascular disease, CNS malignancies, infections or substance abuse.

### Neuropsychological assessment

All participants underwent standardized neuropsychological evaluations. General cognitive function was assessed using the MMSE^23^ and MoCA,^24^ while disease severity was quantified via the CDR scale.^26^ Visuo-abilities were examined using: The Poppelreuter–Ghent Overlapping Figures Test (PGOFT)^27^ for simultaneous perception (identifying overlapping objects). The Rey-Osterrieth Complex Figure Test (ROCFT)^28^ for visuospatial processing. Standardized tests for object/prosopagnosia and colour agnosia. Additional assessments included finger agnosia, right-left disorientation, and apraxia evaluations, consistent with established neurological protocols.^29^

### MRI acquisition

The images were obtained using a 3.0-T hybrid PET/MRI scanner (SIGNA PET/MR, GE Healthcare, WI, USA). A vendor-supplied 19-channel head and neck coil simultaneously acquired PET and MRI data. After each subject was administered ^18^F-FDG (3.7 MBq/kg), three-dimensional T1-weighted (3D-T1) sagittal images and DTI images were acquired during the same session.

The parameters for the multimodal image data were as follows: (i) 3D-T1: The repetition time (TR) was 6.9 ms, echo time (TE) was 2.98 ms, flip angle was 12°, inversion time was 450 ms, matrix size was 256 × 256, field of view (FOV) was 256 × 256 mm^2^, slice thickness was 1 mm and 192 sagittal slices were acquired without gap. The voxel size was 1 × 1 × 1 mm^3^, and the acquisition time was 4 min and 48 s; (ii) DTI: DTI images were obtained using a spin-echo EPI sequence (TR: 16500 ms, TE: 97.6 ms) with b = 1000 s/mm² applied along 30 non-collinear directions. The acquisition parameters included: 70 axial slices (2 mm thickness, no gap), FOV = 220 × 220 mm^2^, matrix = 112 × 112, and 1 excitation.

### Structural network construction and graph theory analysis

The structural connectome analysis pipeline began with diffusion tensor image preprocessing utilizing the PANDA toolbox,^30^ which implements FSL 6.0 algorithms for comprehensive data processing. Initial processing stages involved intracranial tissue extraction from b0 images through the Brain Extraction Tool, followed by correction of eddy current distortions and motion artefacts to optimize data quality. Subsequent tensor estimation generated voxel-level fractional anisotropy maps, while high-resolution T1-weighted images underwent spatial normalization to Montreal Neurological Institute standard space. These transformations enabled precise coregistration of FA maps and inverse warping of the automated anatomical labelling (AAL) atlas into native subject space. We used a deterministic fibre tracking method, the 90 AAL brain regions are the seed region, and the propagation algorithm is FACT (Fiber Assignment by Continuous Tracking), FA threshold is 0.2–1 and angle threshold is 45. A threshold method was used to include real structural connections and avoid spurious ones. Specifically, a threshold value for the streamline number was selected such that the network edges were defined as 1 if the streamline number between two regions was greater than three, and 0 otherwise, as referenced in several previous studies.^31-33^

Graph theoretical characterization was then performed using GRETNA software,^21^ examining network topology across a systematically incremented sparsity range from 0.05 to 0.5 in 0.05 intervals. This analytical approach facilitated computation of multiple network metrics, including global and local efficiency measures, clustering coefficients, characteristic path lengths and organizational features such as assortativity and hierarchy. All topological measures were integrated as area-under-curve values across the specified sparsity range to enable comprehensive network analysis. The imaging analysis was conducted by an experienced neuroradiologist (CSY).

### Statistical analysis

All statistical computations and visualizations were implemented in Python (version 3.11.7). Two-tailed testing was employed throughout the study, with statistical significance defined as *P* < 0.05. Continuous variables demonstrating normal distribution were compared using independent samples *t* tests, while non-parametric Mann–Whitney *U* tests were applied to non-normally distributed data. Partial correlation analyses, controlling for demographic covariates (age, sex, and education level), examined relationships between network metrics and clinical variables. Multiple comparison correction in neuroimaging analyses was performed using false discovery rate (FDR) adjustment, with q < 0.05 considered statistically significant.

## Results

### Demographics

The demographics are shown in Table 1. This study included 34 patients with PCA and 34 healthy controls. The two groups had no significant differences regarding age, gender, or education. The PCA group had an early age of onset, with a mean age of 55.27 ± 4.97 years. MMSE and MoCA scores were lower in the PCA group compared to the healthy controls. The CDR total score was 8.544 ± 2.843. Visual function tests such as PGOFT, ROCFT, finger agnosia test, right–left orientation test, visual–perceptual assessment (object agnosia, prosopagnosia and colour agnosia test), and apraxia test all showed a significant decline in the PCA group compared to the control group.

**Table 1:** Demographic data of the participants

	PCA (*n* = 34)	NC (*n* = 34)	*P*
**Age (years)**	58.882 ± 5.375	59.412 ± 7.203	0.732
**Sex (**male/f**emale)**	11/23	11/23	1
**Education (years)**	10.059 ± 4.625	10.368 ± 3.519	0.758
**Age at onset (years)**	55.265 ± 4.968		
**MMSE**	14.147 ± 4.691	28.647 ± 1.323	<0.001
**MoCA**	7.941 ± 4.256	25.441 ± 2.272	<0.001
**CDR global**	1.515 ± 0.529	0	<0.001
**CDR sum**	8.544 ± 2.843	0	<0.001
**Balint's syndrome**			
**PGOFT**	1.294 ± 1.06	3.971 ± 0.171	<0.001
**ROCFT**	1.676 ± 3.245	14.824 ± 1.66	<0.001
**Gerstmann's syndrome**			
**Finger agnosia test**	2.529 ± 1.44	4	<0.001
**Right-left orientation test**	2.059 ± 1.413	4	<0.001
**Visual-perceptual assessment**			
**Object agnosia test**	2.412 ± 1.861	4.912 ± 0.288	<0.001
**Prosopagnosia test**	5.265 ± 3.396	9.235 ± 1.304	<0.001
**Color agnosia test**	7.324 ± 3.245	9.735 ± 0.567	<0.001
**Apraxia**			
**Apraxia** t**est**	7.897 ± 1.613	9	<0.001

### Comparison of global properties between PCA and NC groups

The PCA group showed significant altered global properties compared to the NC group (Table 2), including decreased Hierarchy (0.494 ± 0.543 versus 0.888 ± 0.356, *P* < 0.001), reduced area under the curve of global efficiency (aEg, 0.192 ± 0.025 versus 0.204 ± 0.007, *P* = 0.007), reduced area under the curve of local efficiency (aEloc, 0.278 ± 0.026 versus 0.298 ± 0.007, *P* < 0.001), reduced area under the curve of the clustering coefficient (aCp, 0.189 ± 0.017 versus 0.201 ± 0.006, *P* < 0.001) and increased area under the curve of the characteristic path length (aLp, 1.089 ± 0.228 versus 0.999 ± 0.035, *P* = 0.027).

**Table 2:** Group comparison of global network properties between PCA and NC groups

	PCA (*n* = 34)	NC (*n* = 34)	Cohen’s *d*	*P*
Assortativity	0.750 ± 0.657	0.799 ± 0.549	−0.0819	0.7367
Hierarchy	0.494 ± 0.543	0.888 ± 0.356	−0.8575	0.0007
aEg	0.192 ± 0.025	0.204 ± 0.007	−0.6747	0.0070
aEloc	0.278 ± 0.026	0.298 ± 0.007	−1.0632	<0.0001
aCp	0.189 ± 0.017	0.201 ± 0.006	−0.8904	0.0005
aLp	1.089 ± 0.228	0.999 ± 0.035	0.5482	0.0271

Abbreviations: aEg, area under the curve of global efficiency; aEloc, area under the curve of local efficiency; aCp, area under the curve of the clustering coefficient; aLp, area under the curve of the characteristic path length; aGamma, area under the curve of the normalized clustering coefficient; aLambda, area under the curve of the normalized characteristic path length; aSigma, area under the curve of small worldness.

### Comparison of nodal properties between PCA and NC

**Figure 1:**
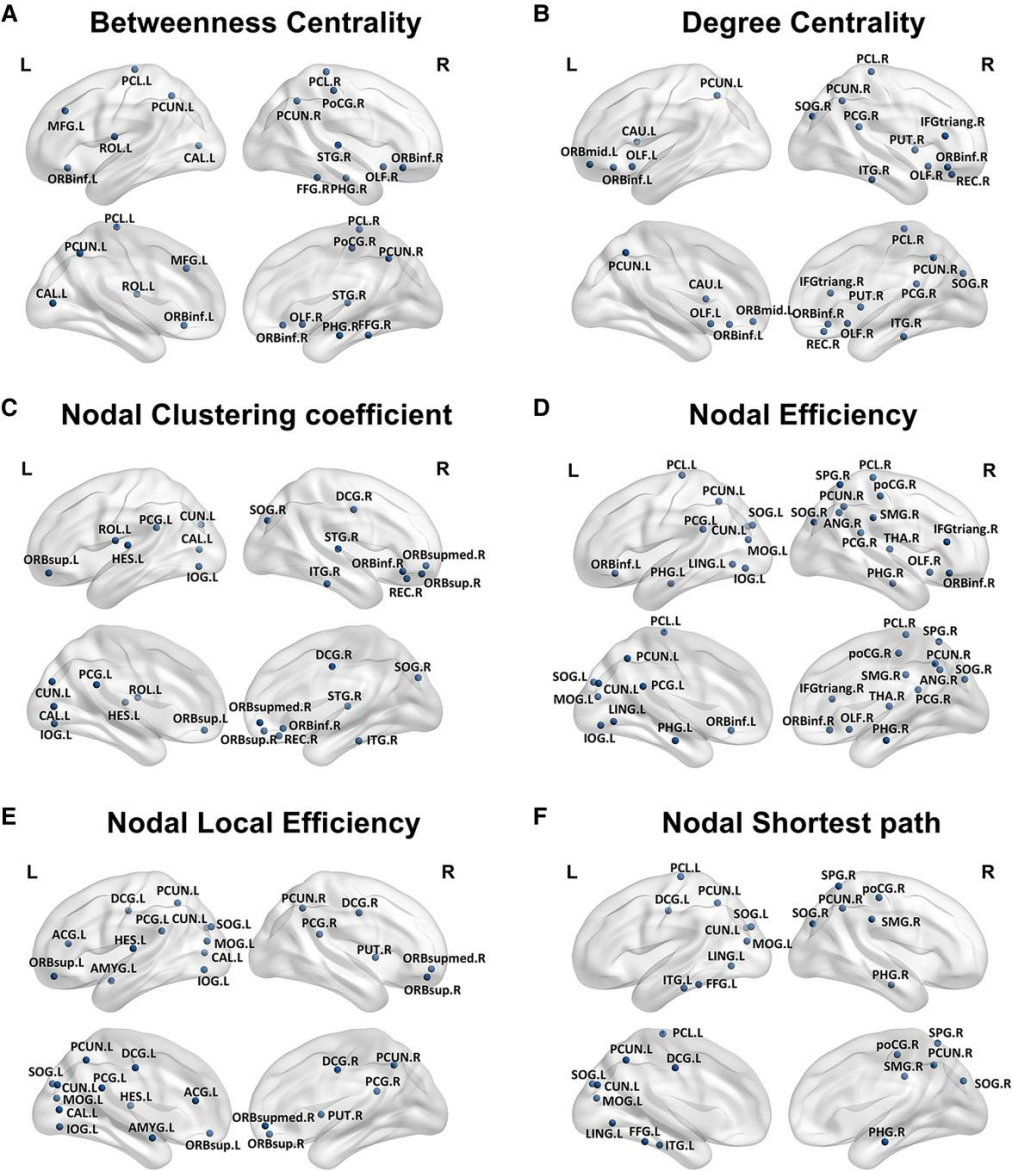
**Alterations of the nodal metrics in PCA.** In the PCA group (*N* = 34), the nodal betweenness centrality. (A), nodal degree centrality (B), nodal clustering coefficient (C), nodal efficiency (D), and nodal local efficiency (E) of nodes in occipital, parietal, temporal, and frontal lobes decreased, while the longer nodal shortest path (F) is mainly in posterior brain regions. T and *P* values for *t*-tests are shown in Supplementary Tables 1–6. Abbreviations: THA.R, right thalamus; STG.R, right superior temporal gyrus; SPG.R, right superior parietal gyrus; SOG.R, right superior occipital gyrus; SOG.L, left superior occipital gyrus; SMG.R, right supramarginal gyrus; ROL.L, left rolandic operculum; REC.R, right rectus gyrus; PUT.R, right putamen; PoCG.R, right postcentral gyrus; PHG.R, right parahippocampal gyrus; PHG.L, left parahippocampal gyrus; PCUN.R, right precuneus; PCUN.L, left precuneus; PCL.R, right paracentral lobule; PCL.L, left paracentral lobule; PCG.R, right posterior cingulate gyrus; PCG.L, left posterior cingulate gyrus; ORBsupmed.R, right superior medial orbitofrontal gyrus; ORBsup.R, right superior orbitofrontal gyrus; ORBsup.L, left superior orbitofrontal gyrus; ORBmid.L, left middle orbitofrontal gyrus; ORBinf.R, right inferior orbitofrontal gyrus; ORBinf.L, left inferior orbitofrontal gyrus; OLF.R, right olfactory cortex; OLF.L, left olfactory cortex; MOG.L, left middle occipital gyrus; MFG.L, left middle frontal gyrus; LING.L, left lingual gyrus; ITG.R, right inferior temporal gyrus; ITG.L, left inferior temporal gyrus; IOG.L, left inferior occipital gyrus; IFGtriang.R, right inferior frontal gyrus triangular part; HES.L, left Heschl's gyrus; FFG.R, right fusiform gyrus; FFG.L, left fusiform gyrus; DCG.R, right dorsal cingulate gyrus; DCG.L, left dorsal cingulate gyrus; CUN.L, left cuneus; CAU.L, left caudate; CAL.L, left calcarine cortex; ANG.R, right angular gyrus; AMYG.L, left amygdala; ACG.L, left anterior cingulate gyrus.

The alteration of nodal properties was illustrated in Fig. 1. Compared with the NC group, the betweenness centrality, degree centrality, nodal clustering coefficient, nodal efficiency and nodal local efficiency were altered in the nodes of the occipital, parietal, temporal and frontal lobes in the PCA group. The longest nodal shortest path is primarily found in the posterior brain regions. Among these node properties, nodal efficiency has the highest number of nodes showing disruption.

### Analyses examining the correlation between global topological features and neuropsychological scales

**Figure 2:**
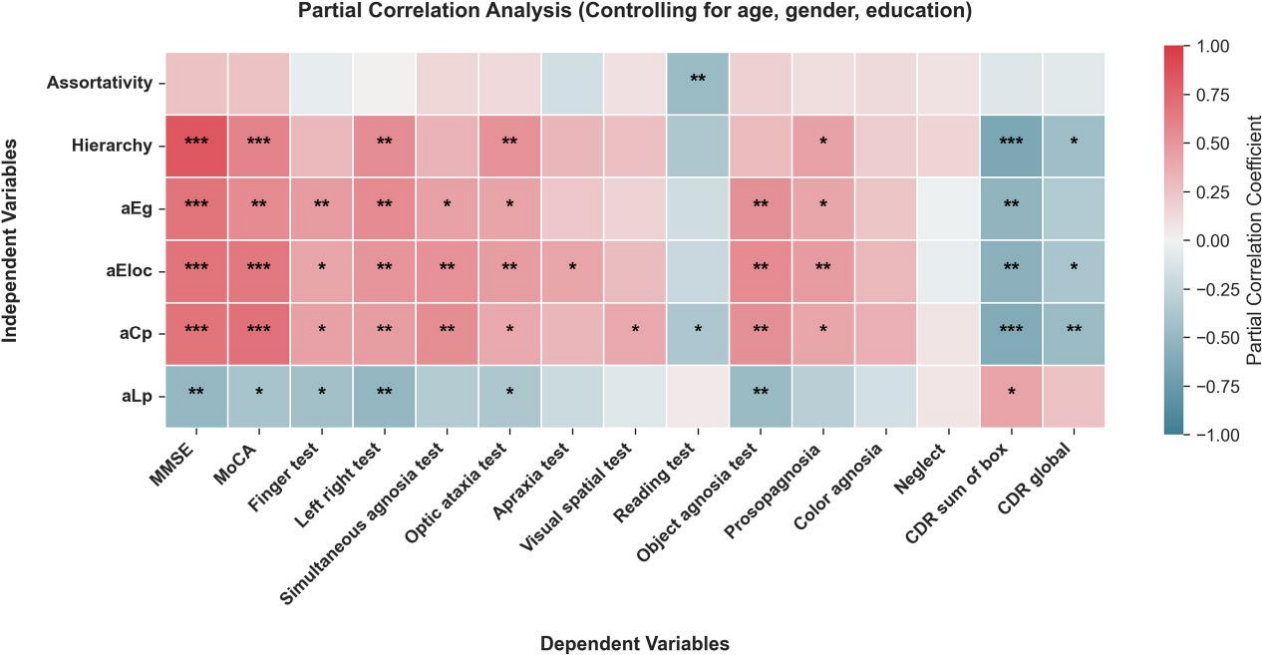
**Partial correlation analysis between network global properties and neuropsychological scales in PCA**. The heatmap illustrates the relationship between global properties of the topological network, including Assortativity, Hierarchy, aEg, aEloc, aCp, aLp, and synchronization with neuropsychological scales in the PCA group (*N* = 34). *R* and *P* values for partial correlation analysis are shown in Supplementary Table 7. *** *P* < 0.001; **P < 0.01; * *P* < 0.05. Abbreviations: PCA, posterior cortical atrophy; aEg, the area under the curve of global efficiency; aEloc, the area under the curve of local efficiency; aCp, the area under the curve of the clustering coefficient; aLp, the area under the curve of the characteristic path length.

The correlation analysis between various global properties and neuropsychological test scores is shown in Table 3 and Fig. 2. The strongest correlations (FDR *P* < 0.001) are observed between MMSE and Hierarchy (*R* = 0.840), MoCA and aCp (*R* = 0.688), and MMSE and aCp (*R* = 0.683). CDR Sum of Boxes consistently shows significant negative correlations with aCp (*R* = −0.615), aEg (*R* = −0.536), aEloc (*R* = −0.559), and Hierarchy (*R* = −0.651). Object Agnosia Test shows significant positive correlations with aCp (*R* = 0.528), aEg (*R* = 0.525) and aEloc (*R* = 0.557).

**Table 3:** Association between global property and neuropsychological scales in PCA

Global property	Neuropsychological test	*R* value	*P* value	FDR adjusted *P*
**aCp**	Visual spatial test	0.391	0.029	0.059
**aCp**	Simultaneous agnosia test	0.537	0.002	0.011
**aCp**	Reading test	−0.377	0.048	0.092
**aCp**	Prosopagnosia	0.411	0.022	0.048
**aCp**	Optic ataxia test	0.392	0.029	0.059
**aCp**	Object agnosia test	0.528	0.002	0.012
**aCp**	MoCA	0.688	<0.001	<0.001
**aCp**	MMSE	0.683	<0.001	<0.001
**aCp**	Left right test	0.456	0.010	0.030
**aCp**	Finger test	0.431	0.015	0.040
**aCp**	CDR sum of box	−0.615	<0.001	0.003
**aCp**	CDR global	−0.483	0.006	0.021
**aEg**	Simultaneous agnosia test	0.439	0.013	0.038
**aEg**	Prosopagnosia	0.408	0.023	0.048
**aEg**	Optic ataxia test	0.429	0.016	0.040
**aEg**	Object agnosia test	0.525	0.002	0.012
**aEg**	MoCA	0.560	0.001	0.008
**aEg**	MMSE	0.685	<0.001	<0.001
**aEg**	Left right test	0.556	0.001	0.008
**aEg**	Finger test	0.463	0.009	0.029
**aEg**	CDR sum of box	−0.536	0.002	0.011
**aEloc**	Simultaneous agnosia test	0.523	0.003	0.012
**aEloc**	Prosopagnosia	0.463	0.009	0.029
**aEloc**	Optic ataxia test	0.457	0.010	0.030
**aEloc**	Object agnosia test	0.557	0.001	0.008
**aEloc**	MoCA	0.658	<0.001	0.001
**aEloc**	MMSE	0.686	<0.001	<0.001
**aEloc**	Left right test	0.503	0.004	0.016
**aEloc**	Finger test	0.417	0.020	0.047
**aEloc**	CDR sum of box	−0.559	0.001	0.008
**aEloc**	CDR global	−0.393	0.029	0.059
**aEloc**	Apraxia test	0.411	0.022	0.048
**aLp**	Optic ataxia test	−0.368	0.042	0.082
**aLp**	Object agnosia test	−0.492	0.005	0.019
**aLp**	MoCA	−0.410	0.022	0.048
**aLp**	MMSE	−0.502	0.004	0.016
**aLp**	Left right test	−0.511	0.003	0.014
**aLp**	Finger test	−0.432	0.015	0.040
**aLp**	CDR sum of box	0.423	0.018	0.043
**Assortativity**	Reading test	−0.481	0.010	0.030
**Hierarchy**	Prosopagnosia	0.436	0.014	0.038
**Hierarchy**	Optic ataxia test	0.521	0.003	0.012
**Hierarchy**	MoCA	0.601	<0.001	0.003
**Hierarchy**	MMSE	0.840	<0.001	<0.001
**Hierarchy**	Left right test	0.543	0.002	0.010
**Hierarchy**	CDR sum of box	−0.651	<0.001	0.001
**Hierarchy**	CDR global	−0.451	0.011	0.032

Abbreviations: aEg, the area under the curve of global efficiency; aEloc, the area under the curve of local efficiency; aCp, the area under the curve of the clustering coefficient; aLp, the area under the curve of the characteristic path length.

### Analyses examining the correlation between visual symptoms and nodal efficiency

**Figure 3:**
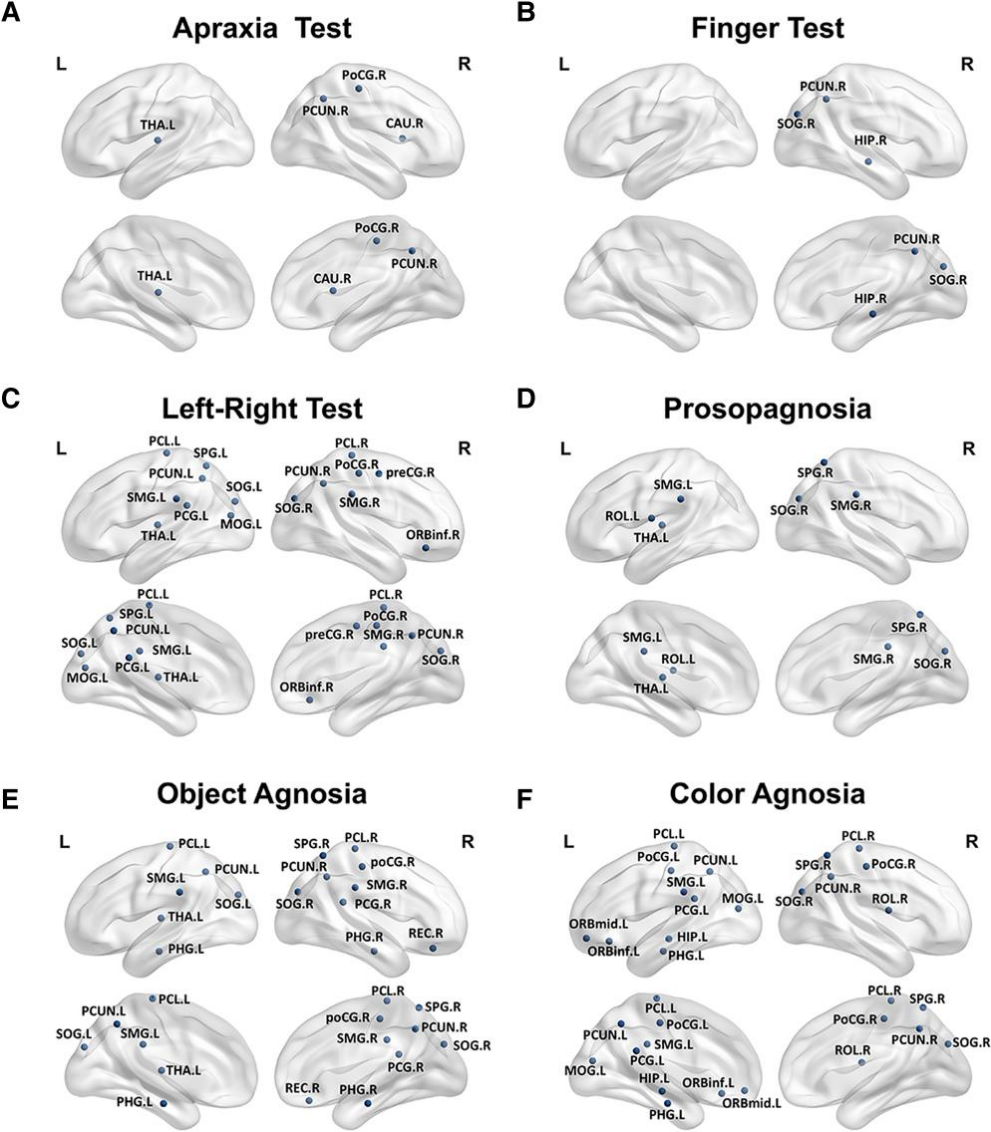
**Correlation analysis between nodal efficiency and assessment scales.** In the PCA group (*N* = 34), the nodal efficiency of nodes associated with apraxia test (A), finger test (B), left-right test (C), prosopagnosia (D), object agnosia (E), and color agnosia (F). *R* and *P* values for partial correlation analysis are shown in Supplementary Table 8. Abbreviations: PoCG.R, right postcentral gyrus; PCUN.R, right precuneus; CAU.R, right caudate; THA.L, left thalamus; ORBmid.L, left orbitofrontal cortex middle; ORBinf.L, left orbitofrontal cortex inferior; ROL.R, right rolandic operculum; PCG.L, left postcentral gyrus; HIP.L, left hippocampus; PHG.L, left parahippocampal gyrus; SOG.R, right superior occipital gyrus; MOG.L, left middle occipital gyrus; PoCG.L, left postcentral gyrus; SPG.R, right superior parietal gyrus; SMG.L, left supramarginal gyrus; PCUN.L, left precuneus; PCL.L, left paracentral lobule; PCL.R, right paracentral lobule; PreCG.R, right precentral gyrus; ORBinf.R, right inferior orbitofrontal cortex; SOG.L, left superior occipital gyrus; MOG.L, left middle occipital gyrus; SPG.L, left superior parietal gyrus; SMG.R, right supramarginal gyrus; THA.L, left thalamus; ROL.L, left rolandic operculum; REC.R, right rectus; PHG.R, right parahippocampal gyrus; SMG.R, right supramarginal gyrus.

We performed a partial correlation analysis between network nodal efficiency and visual symptoms to explore the nodes associated with visual symptoms. The results are shown in Fig. 3. The apraxia test scores are associated with the parietal and subcortical nodes. The finger test is related to the hippocampus, occipital and precuneus nodes. The left–right test is associated with the noes in the frontal (right inferior orbital gyrus), occipital and parietal cortex and thalamus nodes. Prosopagnosia is associated with the occipital, parietal gyrus and thalamus nodes. Object agnosia is associated with frontal (right rectus gyrus), occipital, parietal and thalamic nodes. Colour agnosia is associated with the nodes of the frontal, hippocampus, para-hippocampal, occipital and parietal cortex.

## Discussion

This study is the first to investigate the changes in structural networks in their correlations in PCA patients using graph theory analysis of DTI data. We observed that nodes in the frontal cortex were associated with visual symptoms. This research provides valuable insights into the connectome features and pathophysiological mechanisms of PCA.

### The extent of nodal damage exceeds the focal grey and white matter structural damage

Patients with PCA exhibit more focal grey matter atrophy and hypometabolism in posterior brain regions, according to previous studies.^10,13,14,17,34-42^ As for the alterations of nodal property, the PCA group is affected over a wide range, not limited to the posterior regions but also involving the frontal lobe (inferior frontal, orbital and rectus gyri). The results demonstrate that PCA manifests as a widespread dysregulation of brain network connectivity, where frontal lobe regions actively participate in disease development. Previous studies also provide evidence that the frontal lobe participates in the progression of PCA. The middle frontal and superior frontal gyri were reported as atrophic in an uncorrected threshed.^34^ In another functional MRI topological network analysis, the frontal connections loss was reported to exceed the structural damage in the PCA group.^17^ It is known that the frontal lobes are connected to inferior and superior parietal brain regions by the superior longitudinal fasciculus through the dorsal stream.^43^ In addition, lateral frontal cortices, including the frontal eye field, were key nodes of the dorsal attention network and observed prominent tau uptake in the Aβ+ PCA patients.^16^ Our study provides novel evidence from the structural topological network for the participation of the medial prefrontal cortex in the disease process of PCA.

### Correlation between global properties and neuropsychological scales

Our study is the first to find that in PCA patients, the global topological network properties, including the area under the curve of global efficiency, local efficiency, clustering coefficient, characteristic path length and hierarchy, are correlated with general cognitive status. No prior studies reported the correlation between the structural network and neuropsychological scales in PCA, but some reported the topological network properties in AD. Previous studies have reported that in amnestic mild cognitive impairment patients, the functional topological network path length is correlated with the Auditory Verbal Learning Test,^44^ and in AD, structural network properties are correlated with MMSE and Trail Making Test scores.^33^ These findings indicate the reliability of global topological network properties in reflecting disease states.

### Nodes associated with visual symptoms

We found that the nodal efficiency of the orbital and rectus cortex contributed to the visual symptoms in PCA, indicating a more widespread disrupted pattern for the mechanism of the disease. Raffaella Migliaccio *et al.* found that the frontal connection loss exceeded the structural damage in PCA using functional topological network analysis,^17^ but they did not make a further correlation analysis between the network and visual symptoms. Our findings indicate that different visual symptoms are associated with the specific nodes in the brain network. We found that in our cohort, PCA patients exhibit damage to both the dorsal and ventral streams, leading to overlapping symptoms of agnosia and spatial navigation dysfunction. This is supported by previous studies, which describe that PCA patients primarily show damage to both ventral and dorsal streams involved in visual information processin.^45,46^

Specifically, we found that apraxia is associated with the nodes in the postcentral gyrus, precuneus, and basal ganglia. It is reported that a significant correlation exists between tau deposition in the parietal, temporal and occipital regions of AD and the severity of apraxia.^47^ Our study provides additional evidence for the basal ganglia’s contribution to apraxia. Finger agnosia and left-right disorientation, which are manifestations of Gerstmann syndrome, are primarily caused by damage to the left parietal gyrus,^48^ consistent with the nodal localization in the parietal lobe in our study. Agnosia for objects, faces, and colours is reported to be associated with the ventral stream of the temporal lobe,^49^ but our study found a different distribution pattern, with nodes primarily located in the parietal lobe and only a few nodes distributed in the medial temporal regions, including the hippocampus and parahippocampal gyrus, which is not entirely consistent with previous findings. The inconsistency might be because of the differences in the patient’s cohort and the network analysis method. Notably, some studies have supported a role of the parietal lobe and the dorsal stream in object recognition.^50-53^ However, previous cortical thickness studies have also found that visuoperceptual and visuospatial deficits are not completely independent and exhibit a lot of overlap in their patterns of cortical thinning.^54^ Thus, the dorsal stream might also contribute to agnosia. Previous studies have also shown a significant association between agnosia and reduced perfusion in the orbitofrontal cortex in PCA,^55^ which is not consistent with our findings. In our previous research, we found that the grey matter volume of the left pulvinar is related to object agnosia and prosopagnosia in PCA patients,^29^ and our study also identified the significant role of the left thalamus in prosopagnosia and object agnosia.

When it comes to exploring the correlation between brain regions and visual symptoms, previous studies have shown that left hemineglect is associated with atrophy and hypoperfusion in the right hemisphere, involving large-scale frontoparietal networks.^38^ Simultanagnosia is related to hypometabolism in the right occipital lobe and posterior cingulate gyrus,^6^ and atrophy of white matter also contributes to it.^56^ Optic ataxia is associated with hypometabolism in the left occipital lobe, and oculomotor apraxia is associated with hypometabolism in the left parietal lobe and posterior cingulate gyrus.^6^ We previously observed that simultanagnosia is related to grey matter reduction and decreased functional connectivity in the left middle occipital gyrus and left inferior occipital gyrus in PCA patients.^57^ However, the current study did not find correlations with simultanagnosia, and we did not comprehensively measure hemi-neglect symptoms. Future research should expand the sample size and conduct more systematic measurements to clarify the neural basis of the high-level visual dysfunction in PCA, providing a basis for clinical diagnosis and understanding of the underlying mechanisms.

This study has several limitations. First, the relatively small sample size, attributable to the rarity of PCA, may constrain the generalizability of the findings. Future research should prioritize larger cohorts to validate these results. Second, the cross-sectional design precludes causal inferences regarding the relationship between regional brain changes and visual symptoms. Longitudinal studies are needed to elucidate the temporal dynamics of these alterations and their role in disease progression. Third, there is wide variability in the interpretation of DTI, which is dependent on hardware (type of scanner), software for image processing, and the user who performs the image processing, and age-related white matter degeneration limits reliability in comparing between groups.^58,59^ Fourth, not all healthy controls underwent amyloid-PET, and in the patient group lack of evaluation or documentation of mixed dementia, such as AD with Lewy body co-pathology. Since mixed dementias can mimic PCA in presentation, this oversight may affect diagnostic accuracy and understanding of disease mechanisms. Last, our neuroimaging analysis is based on group-level data, which inherently limits the ability to account for the substantial heterogeneity observed among individual patients.

## Conclusions

This study identified the disruption of the structural network topological pattern in PCA and its clinical relevance. Furthermore, we confirmed that the nodes in the frontal cortex also contribute to visual dysfunction. These results provide novel insights into early diagnosis and understanding of the underlying mechanism of PCA.

## Supplementary Material

fcag014_Supplementary_Data

## Data Availability

The data that support the findings of this study are available from the corresponding author upon reasonable request. The code was shown in the Supplementary material.
