# Supplementary material for: Disrupted brain topological network and its association with clinical features in posterior cortical atrophy
Source: Brain Commun. 2026 Jan 20;8(2):fcag014. doi: 10.1093/braincomms/fcag014 (PMC12971006; doi:10.1093/braincomms/fcag014)
Supplement: fcag014_Supplementary_Data [file fcag014_supplementary_data.zip › Supplementary_material_Code.pdf]

```
import pandas as pd
import numpy as np
import matplotlib.pyplot as plt
import seaborn as sns
import pingouin as pg
import os
from statsmodels.stats.multitest import multipletests

# ======================
# Nature Journal Style Settings
# ======================
plt.style.use('default')
sns.set_style("ticks", {
    "axes.facecolor": "white",
    "figure.facecolor": "white",
    "axes.grid": False,
    "axes.linewidth": 1.0,
    "axes.edgecolor": "black"
})

plt.rcParams.update({
    'font.family': 'Arial',
    'font.size': 10,
    'axes.titlesize': 12,
    'axes.labelsize': 11,
    'xtick.labelsize': 10,
    'ytick.labelsize': 10,
    'figure.dpi': 300,
    'savefig.dpi': 300,
    'figure.figsize': (14, 10),
    'axes.titlepad': 12
})

# ======================
# Data Preparation
# ======================
file_path =
'/Users/chumin/Desktop/python3/PCA/PCA_global_topologcal_scale_correlation_PCAonly
copy.csv'
df = pd.read_csv(file_path)
df.columns = df.columns.str.strip()

independent_vars = ['Assortativity', 'Hierarchy', 'aEg', 'aEloc', 'aCp',
                    'aLp']
dependent_vars = [
    'MMSE',
```

```
    'MoCA',
    'Finger test',
    'Left right test',
    'Simultaneous agnosia test',
    'Optic ataxia test',
    'Apraxia test',
    'Visual spatial test',
    'Reading test',
    'Object agnosia test',
    'Prosopagnosia',
    'Color agnosia',
    'Neglect',
    'CDR sum of box',
    'CDR global'
]
covariates = ['age', 'gender', 'education']

# Initialize matrices
corr_matrix = pd.DataFrame(np.nan, index=independent_vars, columns=dependent_vars)
pvalue_matrix = pd.DataFrame(1.0, index=independent_vars, columns=dependent_vars)
sample_size_matrix = pd.DataFrame(0, index=independent_vars, columns=dependent_vars)

# Calculate partial correlations
for i, ind_var in enumerate(independent_vars):
    for j, dep_var in enumerate(dependent_vars):
        if ind_var not in df.columns or dep_var not in df.columns:
            continue

        df_clean = df[[dep_var, ind_var] + covariates].dropna()
        sample_size_matrix.loc[ind_var, dep_var] = len(df_clean)

        if len(df_clean) < 5:
            continue

        try:
            partial_corr = pg.partial_corr(data=df_clean, x=ind_var, y=dep_var,
                                           covar=covariates, method='pearson')
            corr_matrix.loc[ind_var, dep_var] = partial_corr['r'].values[0]
            pvalue_matrix.loc[ind_var, dep_var] = partial_corr['p-val'].values[0]
        except:
            continue

# ======================
# FDR Correction (within specified scales)
# ======================
# Flatten p-values for FDR correction
```

```
pvals = pvalue_matrix.values.flatten()
mask = ~np.isnan(pvals)
corrected_pvals = np.full_like(pvals, np.nan)
corrected_pvals[mask] = multipletests(pvals[mask], method='fdr_bh')[1]

# Reshape back to matrix
fdr_matrix = pd.DataFrame(corrected_pvals.reshape(pvalue_matrix.shape),
                          index=pvalue_matrix.index,
                          columns=pvalue_matrix.columns)

# =====================
# Generate Comprehensive CSV Output
# =====================
output_dir = '/Users/chumin/Desktop/Heatmap_Results'
os.makedirs(output_dir, exist_ok=True)

# Create long-format dataframe
results = []
for ind_var in independent_vars:
    for dep_var in dependent_vars:
        results.append({
            'Independent_Variable': ind_var,
            'Dependent_Variable': dep_var,
            'R_value': corr_matrix.loc[ind_var, dep_var],
            'P_value': pvalue_matrix.loc[ind_var, dep_var],
            'FDR_adjusted_P': fdr_matrix.loc[ind_var, dep_var],
            'Sample_Size': sample_size_matrix.loc[ind_var, dep_var]
        })

results_df = pd.DataFrame(results)
results_df.to_csv(os.path.join(output_dir, 'Comprehensive_Correlation_Results.csv'),
                  index=False, float_format='%.6f')

# =====================
# Create Heatmap (No Numbers, Bold Labels)
# =====================
plt.figure(figsize=(12, 10))

# Create heatmap without numbers
heatmap = sns.heatmap(
    corr_matrix,
    cmap=sns.diverging_palette(220, 10, as_cmap=True, center="light"),
    center=0,
    annot=False,
    linewidths=0.5,
    linecolor="white",
```

```
    cbar_kws={"shrink": 0.4, "label": "Partial Correlation Coefficient"},
    vmin=-1,
    vmax=1,
    square=True
)

# Add significance markers
for i in range(len(independent_vars)):
    for j in range(len(dependent_vars)):
        pval = pvalue_matrix.iloc[i, j]
        if pval < 0.05:
            if pval < 0.001:
                symbol = '***'
            elif pval < 0.01:
                symbol = '**'
            else:
                symbol = '*'

            plt.text(j + 0.5, i + 0.5, symbol,
                    ha='center', va='center',
                    color='black', fontsize=12, fontweight='bold')

# Customize axis labels with bold font
heatmap.set_xticklabels(heatmap.get_xticklabels(),
                        fontweight='bold',
                        rotation=45,
                        ha='right')
heatmap.set_yticklabels(heatmap.get_yticklabels(),
                        fontweight='bold',
                        rotation=0)

plt.xlabel("Dependent Variables", labelpad=15, fontweight='bold')
plt.ylabel("Independent Variables", labelpad=15, fontweight='bold')
plt.title("Partial Correlation Analysis (Controlling for age, gender, education)",
         pad=20, fontweight='bold')

# Save heatmap
heatmap_path = os.path.join(output_dir, 'Final_Heatmap_BoldLabels.png')
plt.savefig(heatmap_path, bbox_inches='tight', dpi=300)
plt.close()

print("Processing complete!")
print(f"Heatmap saved to: {heatmap_path}")
print(f"Comprehensive results saved to: {os.path.join(output_dir,
'Comprehensive_Correlation_Results.csv')}")
```
