# Supplementary material for: Disrupted brain topological network and its association with clinical features in posterior cortical atrophy
Source: Brain Commun. 2026 Jan 20;8(2):fcag014. doi: 10.1093/braincomms/fcag014 (PMC12971006; doi:10.1093/braincomms/fcag014)
Supplement: fcag014_Supplementary_Data [file fcag014_supplementary_data.zip › Supplementary_tables.docx]

Supplementary Table 1: T and p values for t-tests of group comparison of betweenness centrality (The T and p values in main Figure 1)

| Brain Region | T | P |
| --- | --- | --- |
| MFG.L | -2.1917 | 0.0319 |
| ORBinf.L | 2.3601 | 0.0212 |
| ORBinf.R | 3.3801 | 0.0012 |
| ROL.L | 3.1197 | 0.0027 |
| OLF.R | 2.2439 | 0.0282 |
| PHG.R | 2.6284 | 0.0107 |
| CAL.L | -2.2734 | 0.0263 |
| FFG.R | -2.6468 | 0.0102 |
| PoCG.R | 2.0990 | 0.0396 |
| PCUN.L | 2.2882 | 0.0253 |
| PCUN.R | 2.2675 | 0.0266 |
| PCL.L | 2.3202 | 0.0234 |
| PCL.R | 2.3453 | 0.0220 |
| STG.R | -3.4319 | 0.0010 |

Supplementary Table 2: T and p values for t-tests of group comparison of degree centrality

(The T and p values in main Figure 1)

| Brain Region | T | P |
| --- | --- | --- |
| ORBmid.L | 3.5600 | 0.0007 |
| IFGtriang.R | 2.9335 | 0.0046 |
| ORBinf.L | 3.6570 | 0.0005 |
| ORBinf.R | 3.4074 | 0.0011 |
| ROL.L | 2.2977 | 0.0248 |
| OLF.L | 3.4019 | 0.0011 |
| OLF.R | 3.3132 | 0.0015 |
| SFGmed.R | 2.4158 | 0.0185 |
| ORBsupmed.L | 2.3790 | 0.0203 |
| REC.R | 3.1503 | 0.0025 |
| ACG.R | 2.6267 | 0.0107 |
| PCG.L | 2.4579 | 0.0166 |
| PCG.R | 4.7745 | <0.0001 |
| PHG.R | 2.1299 | 0.0369 |
| SOG.R | 3.0083 | 0.0037 |
| PCUN.L | 3.1721 | 0.0023 |
| PCUN.R | 3.5357 | 0.0007 |
| PCL.L | 2.4091 | 0.0188 |
| PCL.R | 4.2128 | 0.0001 |
| CAU.L | 2.7018 | 0.0088 |
| PUT.R | 2.7297 | 0.0081 |
| STG.R | -2.1188 | 0.0379 |
| ITG.L | 2.0596 | 0.0434 |
| ITG.R | 2.6694 | 0.0096 |

Supplementary Table 3: T and p values for t-tests of group comparison of nodal clustering coefficient (The T and p values in main Figure 1)

| Brain Region | T | P |
| --- | --- | --- |
| ORBsup.L | 3.0936 | 0.0029 |
| ORBsup.R | 3.3191 | 0.0015 |
| ORBinf.R | -2.2009 | 0.0312 |
| ROL.L | -2.5064 | 0.0147 |
| ORBsupmed.R | 2.8039 | 0.0066 |
| REC.R | 2.0750 | 0.0419 |
| DCG.R | 2.9286 | 0.0047 |
| PCG.L | 2.2169 | 0.0301 |
| CAL.L | 2.0063 | 0.0489 |
| CUN.L | 2.2369 | 0.0287 |
| SOG.R | -2.1578 | 0.0346 |
| IOG.L | 2.3844 | 0.0200 |
| HES.L | 3.0853 | 0.0030 |
| STG.R | 2.2288 | 0.0292 |
| ITG.R | -2.2778 | 0.0260 |

Supplementary Table 4: T and p values for t-tests of group comparison of nodal efficiency

(The T and p values in main Figure 1)

| Brain Region | T | P |
| --- | --- | --- |
| ORBmid.L | 2.4313 | 0.0178 |
| IFGtriang.R | 2.7442 | 0.0078 |
| ORBinf.L | 2.7039 | 0.0087 |
| ORBinf.R | 2.8497 | 0.0058 |
| ROL.L | 2.0372 | 0.0456 |
| SMA.L | 2.2528 | 0.0276 |
| OLF.L | 2.2425 | 0.0283 |
| OLF.R | 2.7230 | 0.0083 |
| SFGmed.L | 2.0800 | 0.0414 |
| REC.R | 2.3755 | 0.0204 |
| ACG.R | 2.5058 | 0.0147 |
| DCG.L | 2.1578 | 0.0346 |
| PCG.L | 3.0854 | 0.0030 |
| PCG.R | 3.8643 | 0.0003 |
| HIP.L | 2.3515 | 0.0217 |
| PHG.L | 3.3594 | 0.0013 |
| PHG.R | 3.2145 | 0.0020 |
| CUN.L | 2.6572 | 0.0099 |
| CUN.R | 2.0609 | 0.0433 |
| LING.L | 3.8388 | 0.0003 |
| SOG.L | 3.0364 | 0.0034 |
| SOG.R | 4.5077 | <0.0001 |
| MOG.L | 2.9686 | 0.0042 |
| IOG.L | 3.4502 | 0.0010 |
| FFG.L | 2.2127 | 0.0304 |
| PoCG.R | 4.4366 | <0.0001 |
| SPG.L | 2.4064 | 0.0189 |
| SPG.R | 3.0667 | 0.0031 |
| SMG.L | 2.3124 | 0.0239 |
| SMG.R | 2.8822 | 0.0053 |
| ANG.R | 2.9059 | 0.0050 |
| PCUN.L | 4.3273 | 0.0001 |
| PCUN.R | 4.8249 | <0.0001 |
| PCL.L | 3.4963 | 0.0008 |
| PCL.R | 3.6928 | 0.0005 |
| CAU.L | 2.4023 | 0.0191 |
| THA.L | 2.1193 | 0.0378 |
| THA.R | 2.8054 | 0.0066 |
| ITG.L | 2.6298 | 0.0106 |
| ITG.R | 2.1574 | 0.0346 |

Supplementary Table 5: T and p values for t-tests of group comparison of nodal local efficiency

(The T and p values in main Figure 1)

| Brain Region | T | P |
| --- | --- | --- |
| ORBsup.L | 3.1295 | 0.0026 |
| ORBsup.R | 3.0818 | 0.0030 |
| OLF.L | 2.1650 | 0.0340 |
| ORBsupmed.L | 2.0461 | 0.0447 |
| ORBsupmed.R | 2.7260 | 0.0082 |
| REC.L | 2.0644 | 0.0429 |
| REC.R | 2.0297 | 0.0464 |
| ACG.L | 2.4249 | 0.0181 |
| DCG.L | 2.4111 | 0.0187 |
| DCG.R | 3.5478 | 0.0007 |
| PCG.L | 3.1786 | 0.0023 |
| PCG.R | 2.8625 | 0.0056 |
| AMYG.L | 2.3837 | 0.0200 |
| CAL.L | 3.0806 | 0.0030 |
| CUN.L | 2.6521 | 0.0100 |
| SOG.L | 2.3639 | 0.0210 |
| MOG.L | 2.3983 | 0.0193 |
| IOG.L | 2.4074 | 0.0189 |
| FFG.R | 2.0064 | 0.0489 |
| ANG.R | 2.1869 | 0.0323 |
| PCUN.L | 3.4093 | 0.0011 |
| PCUN.R | 2.3957 | 0.0194 |
| PCL.R | 2.1820 | 0.0327 |
| PUT.R | 2.6214 | 0.0109 |
| PAL.R | 2.0495 | 0.0444 |
| HES.L | 3.1364 | 0.0026 |
| STG.R | 2.1322 | 0.0367 |

Supplementary Table 6 T and p values for t-tests of group comparison of nodal shortest path

(The T and p values in main Figure 1)

| Brain Region | T | P |
| --- | --- | --- |
| PreCG.R | -2.2532 | 0.0276 |
| SMA.L | -2.4386 | 0.0174 |
| DCG.L | -2.6695 | 0.0096 |
| DCG.R | -2.4043 | 0.0190 |
| PHG.R | -3.1639 | 0.0024 |
| CAL.R | -2.0878 | 0.0407 |
| CUN.L | -3.0536 | 0.0033 |
| CUN.R | -2.4018 | 0.0191 |
| LING.L | -3.8776 | 0.0002 |
| SOG.L | -3.1465 | 0.0025 |
| SOG.R | -4.5846 | <0.0001 |
| MOG.L | -3.0699 | 0.0031 |
| FFG.L | -2.8657 | 0.0056 |
| PoCG.R | -4.4832 | <0.0001 |
| SPG.L | -2.5828 | 0.0120 |
| SPG.R | -3.1669 | 0.0023 |
| IPL.L | -2.3968 | 0.0194 |
| SMG.R | -3.0538 | 0.0033 |
| PCUN.L | -4.2706 | 0.0001 |
| PCUN.R | -4.7831 | <0.0001 |
| PCL.L | -3.5538 | 0.0007 |
| ITG.L | -2.8179 | 0.0064 |
| ITG.R | -2.3701 | 0.0207 |

**Abbreviation:** THA.R: Right Thalamus; STG.R: Right Superior Temporal Gyrus; SPG.R: Right Superior Parietal Gyrus; SOG.R: Right Superior Occipital Gyrus; SOG.L: Left Superior Occipital Gyrus; SMG.R: Right Supramarginal Gyrus; ROL.L: Left Rolandic Operculum; REC.R: Right Rectus Gyrus; PUT.R: Right Putamen; PoCG.R: Right Postcentral Gyrus; PHG.R: Right Parahippocampal Gyrus; PHG.L: Left Parahippocampal Gyrus; PCUN.R: Right Precuneus; PCUN.L: Left Precuneus; PCL.R: Right Paracentral Lobule; PCL.L: Left Paracentral Lobule; PCG.R: Right Posterior Cingulate Gyrus; PCG.L: Left Posterior Cingulate Gyrus; ORBsupmed.R: Right Superior Medial Orbitofrontal Gyrus; ORBsup.R: Right Superior Orbitofrontal Gyrus; ORBsup.L: Left Superior Orbitofrontal Gyrus; ORBmid.L: Left Middle Orbitofrontal Gyrus; ORBinf.R: Right Inferior Orbitofrontal Gyrus; ORBinf.L: Left Inferior Orbitofrontal Gyrus; OLF.R: Right Olfactory Cortex; OLF.L: Left Olfactory Cortex; MOG.L: Left Middle Occipital Gyrus; MFG.L: Left Middle Frontal Gyrus; LING.L: Left Lingual Gyrus; ITG.R: Right Inferior Temporal Gyrus; ITG.L: Left Inferior Temporal Gyrus; IOG.L: Left Inferior Occipital Gyrus; IFGtriang.R: Right Inferior Frontal Gyrus Triangular Part; HES.L: Left Heschl's Gyrus; FFG.R: Right Fusiform Gyrus; FFG.L: Left Fusiform Gyrus; DCG.R: Right Dorsal Cingulate Gyrus; DCG.L: Left Dorsal Cingulate Gyrus; CUN.L: Left Cuneus; CAU.L: Left Caudate; CAL.L: Left Calcarine Cortex; ANG.R: Right Angular Gyrus; AMYG.L: Left Amygdala; ACG.L: Left Anterior Cingulate Gyrus

Supplementary Table 7: All results for partial correlation analysis between global property and scales (including R and P values for partial correlation analysis as shown in main Figure 2).

| Variable 1 | Variable 2 | R | P value | FDR djusted P value |
| --- | --- | --- | --- | --- |
| aCp | Visual spatial test | 0.3910 | 0.0290 | 0.0590 |
| aCp | Simultaneous agnosia test | 0.5370 | 0.0020 | 0.0110 |
| aCp | Reading test | -0.3770 | 0.0480 | 0.0920 |
| aCp | Prosopagnosia | 0.4110 | 0.0220 | 0.0480 |
| aCp | Optic ataxia test | 0.3920 | 0.0290 | 0.0590 |
| aCp | Object agnosia test | 0.5280 | 0.0020 | 0.0120 |
| aCp | Neglect | 0.0761 | 0.6839 | 0.7328 |
| aCp | MoCA | 0.6880 | <0.0001 | <0.0001 |
| aCp | MMSE | 0.6830 | <0.0001 | <0.0001 |
| aCp | Left right test | 0.4560 | 0.0100 | 0.0300 |
| aCp | Finger test | 0.4310 | 0.0150 | 0.0400 |
| aCp | Color agnosia | 0.3545 | 0.0504 | 0.0944 |
| aCp | CDR sum of box | -0.6150 | <0.0001 | 0.0030 |
| aCp | CDR global | -0.4830 | 0.0060 | 0.0210 |
| aCp | Apraxia test | 0.3226 | 0.0767 | 0.1279 |
| aEg | Visual spatial test | 0.1645 | 0.3764 | 0.4641 |
| aEg | Simultaneous agnosia test | 0.4390 | 0.0130 | 0.0380 |
| aEg | Reading test | -0.1846 | 0.3470 | 0.4399 |
| aEg | Prosopagnosia | 0.4080 | 0.0230 | 0.0480 |
| aEg | Optic ataxia test | 0.4290 | 0.0160 | 0.0400 |
| aEg | Object agnosia test | 0.5250 | 0.0020 | 0.0120 |
| aEg | Neglect | -0.0163 | 0.9308 | 0.9412 |
| aEg | MoCA | 0.5600 | 0.0010 | 0.0080 |
| aEg | MMSE | 0.6850 | <0.0001 | <0.0001 |
| aEg | Left right test | 0.5560 | 0.0010 | 0.0080 |
| aEg | Finger test | 0.4630 | 0.0090 | 0.0290 |
| aEg | Color agnosia | 0.2434 | 0.1870 | 0.2630 |
| aEg | CDR sum of box | -0.5360 | 0.0020 | 0.0110 |
| aEg | CDR global | -0.3454 | 0.0570 | 0.1027 |
| aEg | Apraxia test | 0.2272 | 0.2191 | 0.3034 |
| aEloc | Visual spatial test | 0.2965 | 0.1053 | 0.1606 |
| aEloc | Simultaneous agnosia test | 0.5230 | 0.0030 | 0.0120 |
| aEloc | Reading test | -0.2204 | 0.2597 | 0.3541 |
| aEloc | Prosopagnosia | 0.4630 | 0.0090 | 0.0290 |
| aEloc | Optic ataxia test | 0.4570 | 0.0100 | 0.0300 |
| aEloc | Object agnosia test | 0.5570 | 0.0010 | 0.0080 |
| aEloc | Neglect | -0.0398 | 0.8315 | 0.8504 |
| aEloc | MoCA | 0.6580 | <0.0001 | 0.0010 |
| aEloc | MMSE | 0.6860 | <0.0001 | <0.0001 |
| aEloc | Left right test | 0.5030 | 0.0040 | 0.0160 |
| aEloc | Finger test | 0.4170 | 0.0200 | 0.0470 |
| aEloc | Color agnosia | 0.3154 | 0.0840 | 0.1349 |
| aEloc | CDR sum of box | -0.5590 | 0.0010 | 0.0080 |
| aEloc | CDR global | -0.3930 | 0.0290 | 0.0590 |
| aEloc | Apraxia test | 0.4110 | 0.0220 | 0.0480 |
| aLp | Visual spatial test | -0.0954 | 0.6097 | 0.6774 |
| aLp | Simultaneous agnosia test | -0.3370 | 0.0638 | 0.1103 |
| aLp | Reading test | 0.0537 | 0.7859 | 0.8225 |
| aLp | Prosopagnosia | -0.3088 | 0.0909 | 0.1436 |
| aLp | Optic ataxia test | -0.3680 | 0.0420 | 0.0820 |
| aLp | Object agnosia test | -0.4920 | 0.0050 | 0.0190 |
| aLp | Neglect | 0.0703 | 0.7071 | 0.7487 |
| aLp | MoCA | -0.4100 | 0.0220 | 0.0480 |
| aLp | MMSE | -0.5020 | 0.0040 | 0.0160 |
| aLp | Left right test | -0.5110 | 0.0030 | 0.0140 |
| aLp | Finger test | -0.4320 | 0.0150 | 0.0400 |
| aLp | Color agnosia | -0.1647 | 0.3761 | 0.4641 |
| aLp | CDR sum of box | 0.4230 | 0.0180 | 0.0430 |
| aLp | CDR global | 0.2604 | 0.1572 | 0.2263 |
| aLp | Apraxia test | -0.2055 | 0.2674 | 0.3592 |
| Assortativity | Visual spatial test | 0.1015 | 0.5868 | 0.6601 |
| Assortativity | Simultaneous agnosia test | 0.1512 | 0.4167 | 0.5000 |
| Assortativity | Reading test | -0.4810 | 0.0100 | 0.0300 |
| Assortativity | Prosopagnosia | 0.1167 | 0.5317 | 0.6135 |
| Assortativity | Optic ataxia test | 0.1401 | 0.4521 | 0.5354 |
| Assortativity | Object agnosia test | 0.1842 | 0.3212 | 0.4190 |
| Assortativity | Neglect | 0.0925 | 0.6207 | 0.6813 |
| Assortativity | MoCA | 0.2596 | 0.1584 | 0.2263 |
| Assortativity | MMSE | 0.2610 | 0.1561 | 0.2263 |
| Assortativity | Left right test | 0.0085 | 0.9637 | 0.9637 |
| Assortativity | Finger test | -0.0470 | 0.8019 | 0.8296 |
| Assortativity | Color agnosia | 0.1278 | 0.4934 | 0.5767 |
| Assortativity | CDR sum of box | -0.1057 | 0.5714 | 0.6509 |
| Assortativity | CDR global | -0.0857 | 0.6468 | 0.7014 |
| Assortativity | Apraxia test | -0.1770 | 0.3408 | 0.4382 |
| Hierarchy | Visual spatial test | 0.2663 | 0.1477 | 0.2215 |
| Hierarchy | Simultaneous agnosia test | 0.3432 | 0.0587 | 0.1036 |
| Hierarchy | Reading test | -0.3686 | 0.0536 | 0.0985 |
| Hierarchy | Prosopagnosia | 0.4360 | 0.0140 | 0.0380 |
| Hierarchy | Optic ataxia test | 0.5210 | 0.0030 | 0.0120 |
| Hierarchy | Object agnosia test | 0.2992 | 0.1021 | 0.1584 |
| Hierarchy | Neglect | 0.1603 | 0.3891 | 0.4732 |
| Hierarchy | MoCA | 0.6010 | <0.0001 | 0.0030 |
| Hierarchy | MMSE | 0.8400 | <0.0001 | <0.0001 |
| Hierarchy | Left right test | 0.5430 | 0.0020 | 0.0100 |
| Hierarchy | Finger test | 0.3178 | 0.0815 | 0.1333 |
| Hierarchy | Color agnosia | 0.2010 | 0.2783 | 0.3683 |
| Hierarchy | CDR sum of box | -0.6510 | <0.0001 | 0.0010 |
| Hierarchy | CDR global | -0.4510 | 0.0110 | 0.0320 |
| Hierarchy | Apraxia test | 0.3269 | 0.0726 | 0.1233 |

**Abbreviation:** PCA, posterior cortical atrophy; aEg, the area under the curve of global efficiency; aEloc, the area under the curve of local efficiency; aCp, the area under the curve of the clustering coefficient; aLp, the area under the curve of the characteristic path length.

Supplementary Table 8: All Results for partial correlation analysis between nodal efficiency and scales (including R and P values for partial correlation analysis as shown in main Figure 3).

| Variable 1 | Variable 2 | R | P value |
| --- | --- | --- | --- |
| Apraxia test | CAU.R | -0.3277 | 0.0077 |
| Apraxia test | PCUN.R | -0.2900 | 0.0191 |
| Apraxia test | PoCG.R | -0.2883 | 0.0198 |
| Apraxia test | THA.L | -0.2894 | 0.0194 |
| Color agnosia | HIP.L | -0.3492 | 0.0044 |
| Color agnosia | MOG.L | -0.2803 | 0.0237 |
| Color agnosia | ORBinf.L | -0.3165 | 0.0102 |
| Color agnosia | ORBmid.L | -0.3482 | 0.0045 |
| Color agnosia | PCG.L | -0.3116 | 0.0115 |
| Color agnosia | PCL.L | -0.3189 | 0.0096 |
| Color agnosia | PCL.R | -0.3379 | 0.0059 |
| Color agnosia | PCUN.L | -0.3039 | 0.0138 |
| Color agnosia | PCUN.R | -0.3366 | 0.0061 |
| Color agnosia | PHG.L | -0.2899 | 0.0192 |
| Color agnosia | PoCG.L | -0.3110 | 0.0117 |
| Color agnosia | PoCG.R | -0.4566 | 0.0001 |
| Color agnosia | ROL.R | -0.2823 | 0.0227 |
| Color agnosia | SMG.L | -0.3339 | 0.0066 |
| Color agnosia | SOG.R | -0.3692 | 0.0025 |
| Color agnosia | SPG.R | -0.3012 | 0.0147 |
| Finger test | HIP.R | -0.2837 | 0.0220 |
| Finger test | PCUN.R | -0.3022 | 0.0144 |
| Finger test | SOG.R | -0.3069 | 0.0129 |
| Left right test | MOG.L | -0.3612 | 0.0031 |
| Left right test | ORBinf.R | -0.2732 | 0.0277 |
| Left right test | PCG.L | -0.3181 | 0.0098 |
| Left right test | PCL.L | -0.3063 | 0.0131 |
| Left right test | PCL.R | -0.3325 | 0.0068 |
| Left right test | PCUN.L | -0.2883 | 0.0199 |
| Left right test | PCUN.R | -0.3691 | 0.0025 |
| Left right test | PoCG.R | -0.4293 | 0.0004 |
| Left right test | PreCG.R | -0.2751 | 0.0265 |
| Left right test | SMG.L | -0.275 | 0.0266 |
| Left right test | SMG.R | -0.2838 | 0.022 |
| Left right test | SOG.L | -0.3014 | 0.0147 |
| Left right test | SOG.R | -0.4183 | 0.0005 |
| Left right test | SPG.L | -0.2819 | 0.0229 |
| Left right test | THA.L | -0.3247 | 0.0083 |
| Object agnosia | PCL.L | -0.3566 | 0.0036 |
| Object agnosia | PCL.R | -0.4250 | 0.0004 |
| Object agnosia | PCUN.L | -0.4358 | 0.0003 |
| Object agnosia | PCUN.R | -0.5255 | <0.0001 |
| Object agnosia | PHG.L | -0.3663 | 0.0027 |
| Object agnosia | PHG.R | -0.4350 | 0.0003 |
| Object agnosia | PoCG.R | -0.4553 | 0.0001 |
| Object agnosia | REC.R | -0.2914 | 0.0185 |
| Object agnosia | SMG.L | -0.3083 | 0.0125 |
| Object agnosia | SMG.R | -0.2910 | 0.0187 |
| Object agnosia | SOG.L | -0.3401 | 0.0056 |
| Object agnosia | SOG.R | -0.5221 | <0.0001 |
| Object agnosia | SPG.L | -0.3036 | 0.0139 |
| Object agnosia | SPG.R | -0.3597 | 0.0033 |
| Object agnosia | THA.L | -0.3645 | 0.0028 |
| Object agnosia | THA.R | -0.3052 | 0.0134 |
| Object agnosia | TPOmid.R | -0.2720 | 0.0284 |
| Prosopagnosia | ROL.L | -0.3033 | 0.0141 |
| Prosopagnosia | SMG.L | -0.3573 | 0.0035 |
| Prosopagnosia | SMG.R | -0.2845 | 0.0216 |
| Prosopagnosia | SOG.R | -0.3954 | 0.0011 |
| Prosopagnosia | SPG.R | -0.3460 | 0.0048 |
| Prosopagnosia | THA.L | -0.2784 | 0.0247 |

**Abbreviation:** PoCG.R: Right Postcentral Gyrus; PCUN.R: Right Precuneus; CAU.R: Right Caudate; THA.L: Left Thalamus; ORBmid.L: Left Orbitofrontal Cortex Middle; ORBinf.L: Left Orbitofrontal Cortex Inferior; ROL.R: Right Rolandic Operculum; PCG.L: Left Postcentral Gyrus; HIP.L: Left Hippocampus; PHG.L: Left Parahippocampal Gyrus; SOG.R: Right Superior Occipital Gyrus; MOG.L: Left Middle Occipital Gyrus; PoCG.L: Left Postcentral Gyrus; SPG.R: Right Superior Parietal Gyrus; SMG.L: Left Supramarginal Gyrus; PCUN.L: Left Precuneus; PCL.L: Left Paracentral Lobule; PCL.R: Right Paracentral Lobule; PreCG.R: Right Precentral Gyrus; ORBinf.R: Right Orbitofrontal Cortex Inferior; SOG.L: Left Superior Occipital Gyrus; MOG.L: Left Middle Occipital Gyrus; SPG.L: Left Superior Parietal Gyrus; SMG.R: Right Supramarginal Gyrus; THA.L: Left Thalamus; ROL.L: Left Rolandic Operculum; REC.R: Right Recall; PHG.R: Right Parahippocampal Gyrus; SMG.R: Right Supramarginal Gyrus
